# Bi-Directional and Operand-Controllable In-Memory Computing for Boolean Logic and Search Operations with Row and Column Directional SRAM (RC-SRAM)

**DOI:** 10.3390/mi15081056

**Published:** 2024-08-22

**Authors:** Han Xiao, Ruiyong Zhao, Yulan Liu, Yuanzhen Liu, Jing Chen

**Affiliations:** 1 Shanghai Institute of Microsystem and Information Technology, Chinese Academy of Sciences, Shanghai 200031, China; hanx@mail.sim.ac.cn (H.X.); zry@mail.sim.ac.cn (R.Z.); ylliu@mail.sim.ac.cn (Y.L.); liuyuanzhen23@mails.ucas.ac.cn (Y.L.); 2 University of Chinese Academy of Sciences, Beijing 100049, China

**Keywords:** in-memory computing, SRAM, content-addressable memory, Boolean logic operation

## Abstract

The von Neumann architecture is no longer sufficient for handling large-scale data. In-memory computing has emerged as the potent method for breaking through the memory bottleneck. A new 10T SRAM bitcell with row and column control lines called RC-SRAM is proposed in this article. The architecture based on RC-SRAM can achieve bi-directional and operand-controllable logic-in-memory and search operations through different signal configurations, which can comprehensively respond to various occasions and needs. Moreover, we propose threshold-controlled logic gates for sensing, which effectively reduces the circuit area and improves accuracy. We validate the RC-SRAM with a 28 nm CMOS technology, and the results show that the circuits are not only full featured and flexible for customization but also have a significant increase in the working frequency. At VDD = 0.9 V and T = 25 °C, the bi-directional search frequency is up to 775 MHz and 567 MHz, and the speeds for row and column Boolean logic reach 759 MHz and 683 MHz.

## 1. Introduction

With the advancements in machine learning and artificial intelligence, the von Neumann architecture is no longer adequate for handling large-scale data [1]. Frequent data transfers between memory units and arithmetic logic units (ALUs) lead to increased latency and unnecessary power consumption.

In-memory computing (IMC) provides an effective solution for alleviating the von Neumann bottleneck by enabling memory to process data [2,3]. Currently, IMC has been implemented using various memory types, including DRAM [4,5], SRAM, and non-volatile memory (NVM) [6,7,8]. SRAM is particularly noteworthy for its outstanding performance, characterized by low power consumption and high process compatibility. IMC based on SRAM primarily involves operations such as multiply–accumulate (MAC) [9,10], multi-bit multiplication [11,12], and Boolean logic, with a key focus on logic-in-memory operations in this paper.

The fundamental approach for implementing Boolean logic operations is to sense the voltage on bit lines using sensitive amplifiers (SAs) to obtain the results [13]. Customized SA can further enhance the sensing efficiency and calculation accuracy [14]. Additionally, a multi-functional IMC architecture can be achieved by designing new bitcell and peripheral auxiliary circuits. A 10T bitcell proposed in [15] with bi-directional read ports, accompanied by a self-terminating structure, can support both Boolean logic and content-addressable memory (CAM) search operations. Moreover, the author in [16] proposed a self-cycling 8T bitcell capable of executing both Boolean logic and copy operations.

However, many existing IMC architectures are limited in reconfigurability and flexibility, as they typically support fixed-bit and unidirectional operations. For instance, architectures referenced in [17,18] are constrained to performing only two-input logic operations, and those mentioned in [19] are likewise restricted to handling four-input logic operations. Furthermore, all architectures mentioned in [18,19] can only perform logic operations in one direction. Similar to Boolean logic, most CAM operations are primarily unidirectional searches conducted across the entire array [18,19]. They lack the capability to customize the number of rows or columns for searching or to support bi-directional searches. While an architecture described in [15] does provide bi-directional capabilities, its CAM search is limited to full-array searches, and its row-wise OR/NOR and column-wise AND/NAND operations require additional self-termination structures to achieve bit customization.

In this paper, we propose a new type of SRAM called Row and Column Directional SRAM (RC-SRAM) specifically designed for Boolean logic and CAM search operations. In the RC-SRAM structure, the gates of N5 and N6 are connected to Q/QB, which effectively isolates the internal nodes of the bitcell from external circuits. In IMC mode, the proposed RC-SRAM, in combination with auxiliary circuits and various signal configuration methods, enables bi-directional operations and allows for operand-defined logic and search operations. This significantly enhances the flexibility and reconfigurability of IMC architectures. Moreover, by replacing traditional SAs and analog-to-digital converters (ADCs) with threshold-controllable logic gates, the architecture reduces the circuit area requirements while improving sensing efficiency.

This paper is structured as follows: Section 2 outlines the RC-SRAM bitcell and provides an overview of the IMC architecture. In Section 3, the paper elaborates on the working principle and specific process of the SRAM mode, Boolean logic mode, and CAM search mode. Section 4 presents a comprehensive analysis of the experimental results, and Section 5 offers concluding remarks on the work presented in this paper.

## 2. RC-SRAM and IMC Architecture

**Figure 1:**
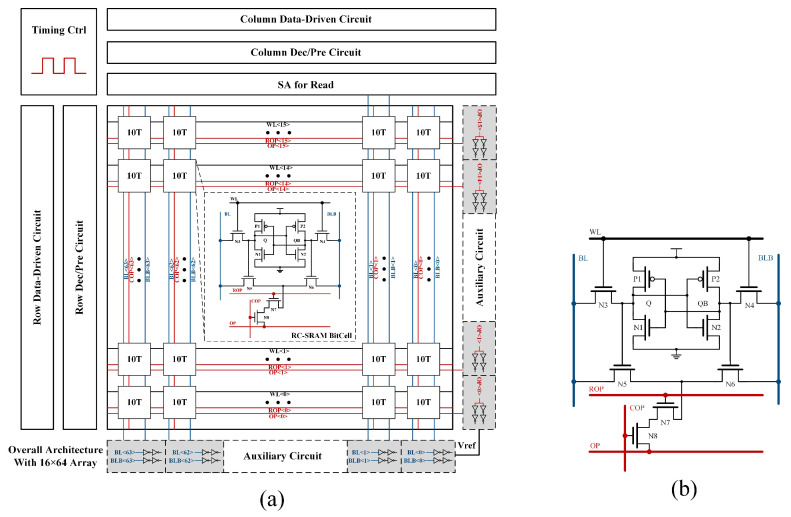
(**a**) The RC-SRAM-based IMC architecture. (**b**) Schematic of RC-SRAM bitcell.

The schematic of RC-SRAM is illustrated in Figure 1b. RC-SRAM extends the 6T bitcell by incorporating four transistors. N5 and N6 serve as data-control transistors, with their gate individually connected to Q and QB. N7 and N8 control the direction of operations, with their gate respectively connected to the row-sharing line ROP and column-sharing line COP. Additionally, Q/QB, ROP, and COP control the formation of the charge/discharge path between BL/BLB and the row-sharing line OP by regulating the on/off states of N5–N8, thus achieving a bi-directional, operand-controlled IMC mode.

The proposed RC-SRAM-based IMC architecture is shown in Figure 1a. The architecture primarily comprises a 1Kb RC-SRAM array, timing control circuits, data driver circuits, pre-charge and pre-discharge modules, row/column decoding circuits, and peripheral auxiliary circuits.

## 3. Operating Mode and Working Principle

### 3.1. SRAM Mode

**Figure 2:**
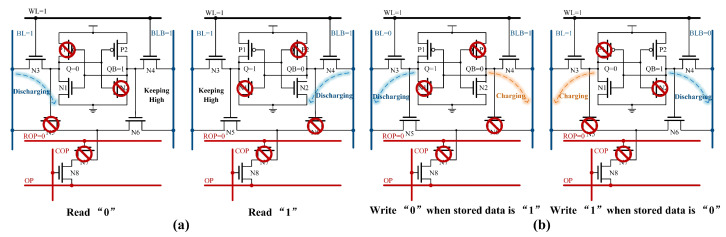
(**a**) The RC-SRAM bitcell state of read operation. (**b**) The RC-SRAM bitcell state of write operation.

Figure 2 illustrates the state of the RC-SRAM bitcell during read and write operations. In SRAM mode, a scenario can occur where both N7 and N8 are on because of the uncertain voltage levels on ROP and COP before read and write operations. At this time, there is a connection established between BL/BLB and OP, resulting in increased power consumption during BL/BLB pre-charging. It becomes more difficult for WDATA to drive the BL/BLB during write operations, and for the BL/BLB to read the stored data during read operations. To address this issue, a ROP pre-discharge process is introduced at the onset of the read/write operation, preventing the formation of any path between OP and BL/BLB.

### 3.2. Boolean Logic Mode

The RC-SRAM proposed in this paper is configured with a row-wise operation signal ROP and a column-wise operation signal COP. The customization of operation bits and the bi-directional operation are achieved through the use of ROP and COP, which control the on/off states of N7 and N8. In addition, the threshold-controllable logic gates replace traditional SAs and ADCs to complete the parallel output of six Boolean logic operations.

#### 3.2.1. Row-Wise Boolean Logic Operation

Row-wise Boolean logic operation means that, within a single cycle, selected rows in each column of the array perform the Boolean logic operations and output all the results of the operations in parallel through the auxiliary circuits.

When the control signal is enabled, all ROPs are pre-discharged, and all COPs are pre-charged. Subsequently, the bit lines are pre-charged to a high state, and all OPs are grounded. Once the signals are set up, the address decoder decodes the involved rows and initiates the charging of their ROPs to commence the operation.

**Figure 3:**
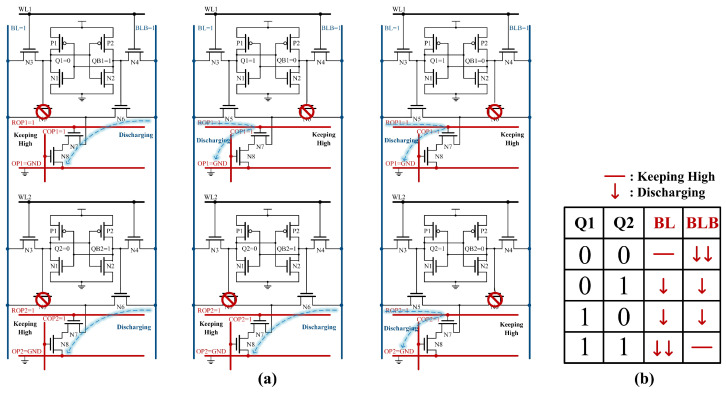
(**a**) The RC-SRAM bitcell state of row-wise Boolean logic operation. (**b**) Discharge conditions of BL and BLB for different state.

Figure 3a displays three cases of Boolean operations with two rows of cells. Figure 3b shows the discharge conditions of BL/BLB for different Q1 and Q2. If both Q1 and Q2 are 0, BL cannot form a path with OP and thus remains high. When either Q1 or Q2 equals 1, N5 in the corresponding bitcell is on, allowing BL to establish a path with OP, resulting in the discharge of BL. Similarly, the formation of the discharge path between BLB and OP correlates with the Q value.

**Figure 4:**
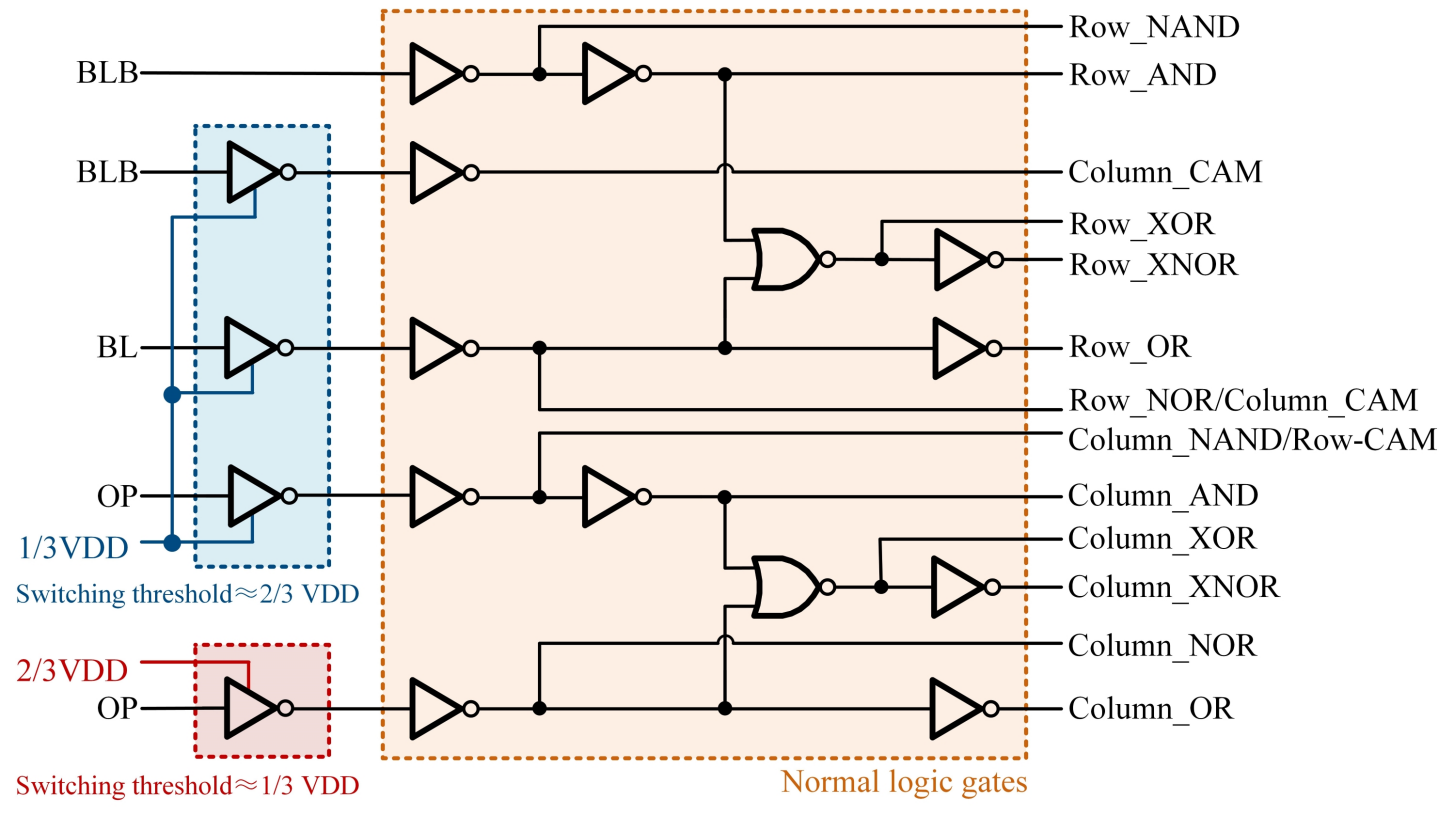
Auxiliary circuits for sensing.

Additional auxiliary circuits are required for sensing to obtain the outputs as shown in Figure 4. The results of the AND/NAND operations are obtained by sensing BLB through a two-stage common inverter gate (INV). The BL is sensed by an INV with the threshold of about 2/3 VDD to classify the case where the operands are all 0 versus the presence of 1 in the operands, and then the output is corrected to a standard digital level by a two-stage INV to obtain the result of the OR/NOR operation. The choice of a 2/3 VDD threshold voltage is based on the fact that the BL discharges relatively slowly, and a higher threshold voltage results in faster sensing. Compared to traditional SAs and ADCs, threshold-controllable gate circuits reduce circuit area, lower delays, and maintain output accuracy with the fault tolerance.

**Figure 5:**
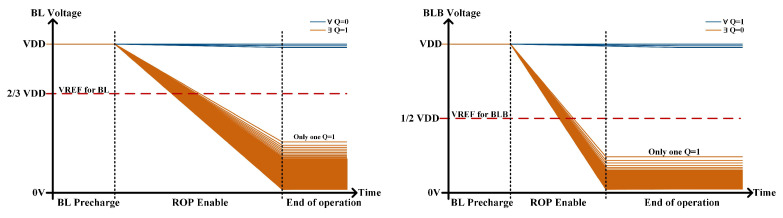
BL/BLB voltage of row-wise Boolean logic operation through transient simulations.

To ensure the accuracy of arithmetic results, it is imperative to consider all potential scenarios where the number of operational bits ranges from 2 to 64. This corresponds to the possible existence of 0 to 64 discharge paths between BL/BLB and OP. Figure 5 illustrates the voltage variation of BL/BLB through transient simulations conducted for various scenarios. It can be observed that the auxiliary circuits ensure operational accuracy in all scenarios, provided that it can reliably sense cases where only one Q is 1.

#### 3.2.2. Column-Wise Boolean Logic Operation

For column-wise Boolean logic operations, when the control signal is enabled, all ROPs are pre-charged, and all COPs are pre-discharged. Subsequently, all OPs are pre-charged to 1/2 VDD, while BL is connected to VDD, and BLB is connected to the ground. Once the signals are set up, the address decoder decodes the involved columns and initiates the charging of their COPs to commence the operation.

**Figure 6:**
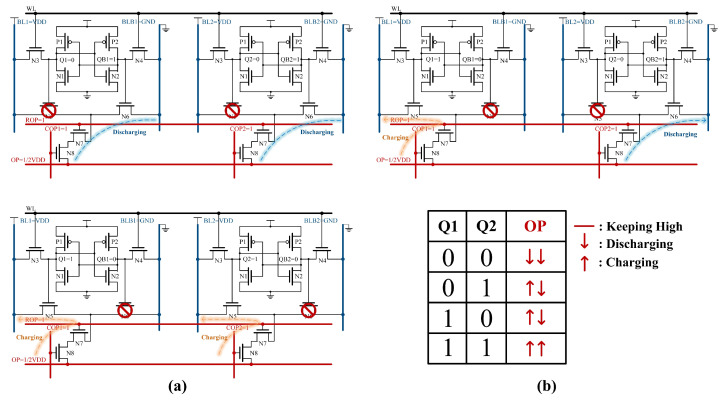
(**a**) The RC-SRAM bitcell state of column-wise Boolean logic operation. (**b**) Discharge or charge conditions of OP for different state.

As shown in Figure 6a, this schematic diagram illustrates Boolean operations with two columns of bit cells. Figure 6b shows the discharge or charge conditions of the OP for various values of Q1 and Q2. When both Q1 and Q2 are 0, N5s are off, and N6s are on, forming two discharging paths between OP and BLB. When Q1 is 1 and Q2 is 0, a charging path to BL exists alongside a discharging path to BLB for OP. In addition, when both Q1 and Q2 are 1, two charging paths are established between OP and BL.

The column-wise Boolean logic operations also require auxiliary circuits as shown in Figure 4 to obtain the results. The OP is sensed by an INV with a threshold voltage of 2/3 VDD, distinguishing between cases where Q is all 1 and others. Additionally, the OP is sensed by an INV with a threshold voltage of 1/3 VDD, distinguishing between cases where Q is all 0 and others.

To ensure the accuracy of the calculation results, it is imperative to consider all potential scenarios where the number of operational bits ranges from 2 to 16.

**Figure 7:**
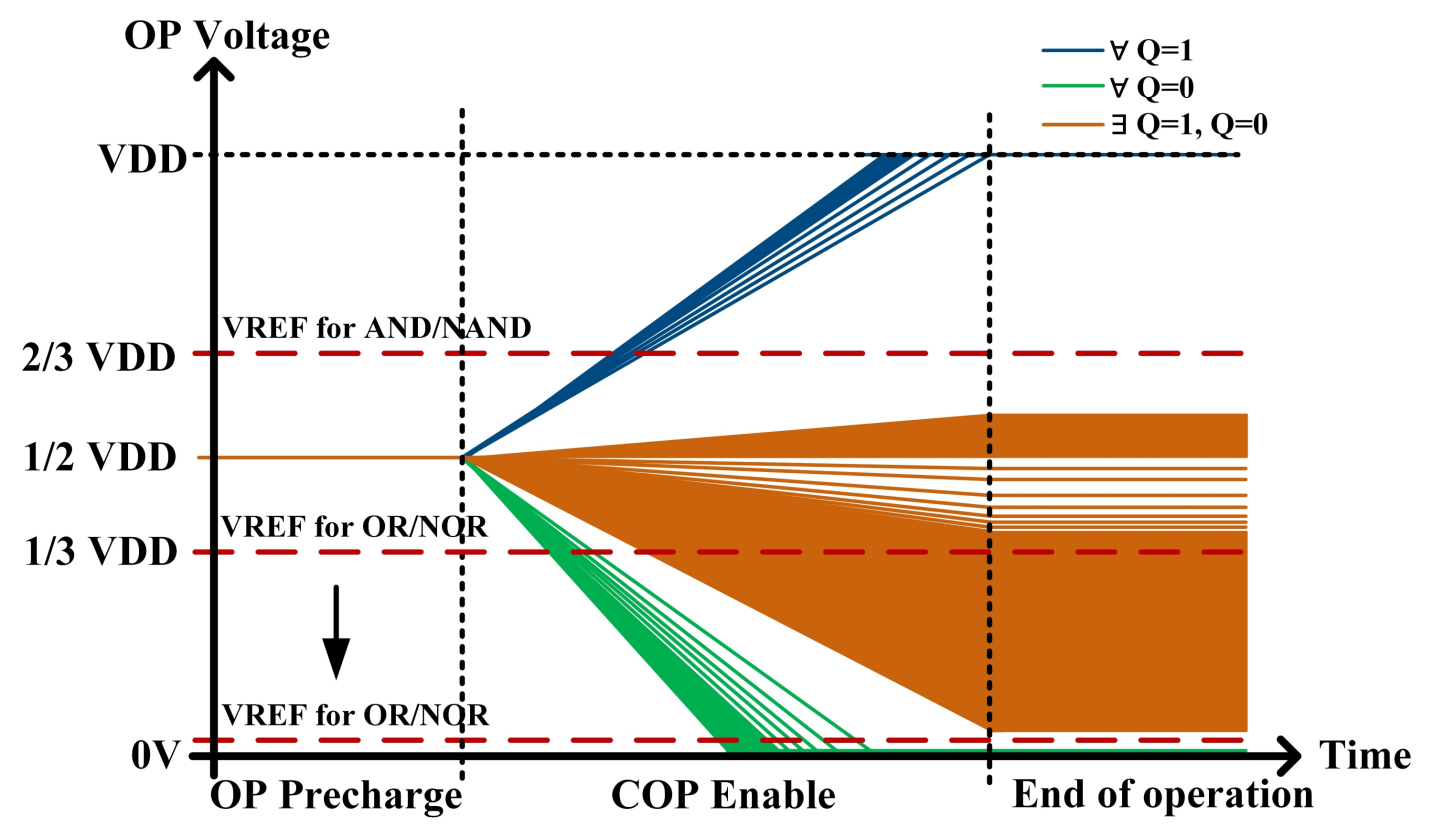
OP voltage of column-wise Boolean logic operation through transient simulations.

Unlike the row-wise Boolean logic operation, an increase in the number of operation bits results in a change in the voltage of the OP as illustrated in Figure 7. Since the OP discharging capability is much larger than the charging capability in the designed circuit, the INV for OR/NOR must consistently lower its threshold in order to differentiate the case where Q is all zero. It has been observed that as the number of operands increases, the task of distinguishing the scenario where Q is all zeros becomes progressively more challenging. To ensure result accuracy, it is advisable to limit the number of operands to fewer bits.

### 3.3. CAM Mode

Based on the search accuracy, CAM can be divided into BCAM and TCAM. The search implemented in this paper is BCAM, which achieves accurate matching by comparing the stored data with the search data. Due to the special RC-SRAM structure, the search mode offers the flexibility to customize both the number of search rows and columns, as well as enabling bi-directional searching by modifying the signal configuration.

#### 3.3.1. Row-Wise CAM Operation

Row-wise CAM means that the search data are compared in parallel with each row of data stored in the array. When the control signal is enabled, all ROPs are pre-charged, and all COPs are pre-discharged. Subsequently, the OP is pre-charged and employed as a match line, while BL and BLB are designated as search lines, with their voltages configured based on the search data. When the search data are 1, BL/BLB is set to 1/0. Conversely, when the search data are 0, BL/BLB is set to 0/1.

**Figure 8:**
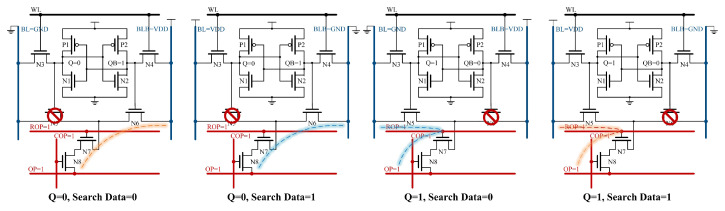
The RC-SRAM bitcell state of row-wise search operation.

For a single RC-SRAM illustrated in Figure 8, when Q is 0 and the search data are also 0, OP will form a charging path with BLB through N6, N7, and N8. When Q is 0 and the search data are 1, OP will form a discharging path with BLB through N6, N7, and N8. When Q is 1 and the search data are 0, OP will form a discharging path with BL through N5, N7, and N8. When Q is 1 and the search data are also 1, OP will form a charging path with BL through N5, N7, and N8.

**Figure 9:**
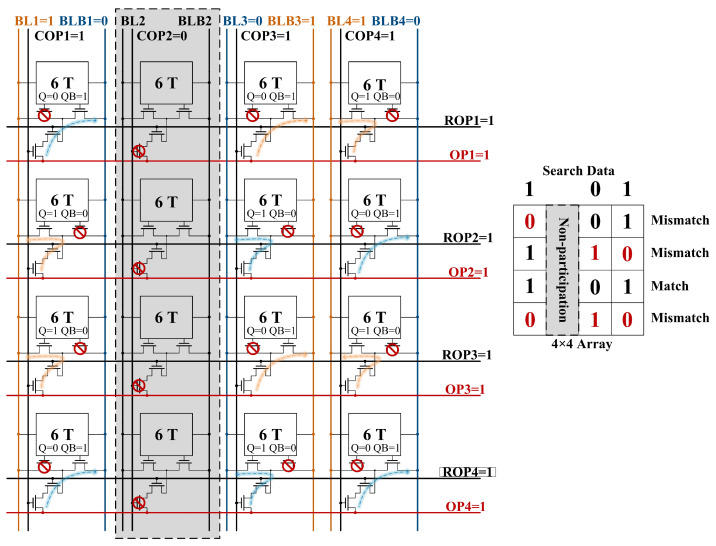
Example of row-wise search operation in 4 × 4 array.

As illustrated in Figure 9, the proposed row-wise search process is demonstrated using a simplified 4 × 4 array. In the context of the customized search operation, columns 1, 3, and 4 are designated for the search process, while column 2 is not involved in the search. Consequently, COP2 is set to 0, indicating that it is not part of the search operation, and the remaining COPs are pre-charged for the search. During the search process, it is identified that only the data stored in the third row match the search criteria “101”, while the data in the other rows do not align with the specified search data. Successful matching occurs when all paths of the OP are fully connected at a high level.

**Figure 10:**
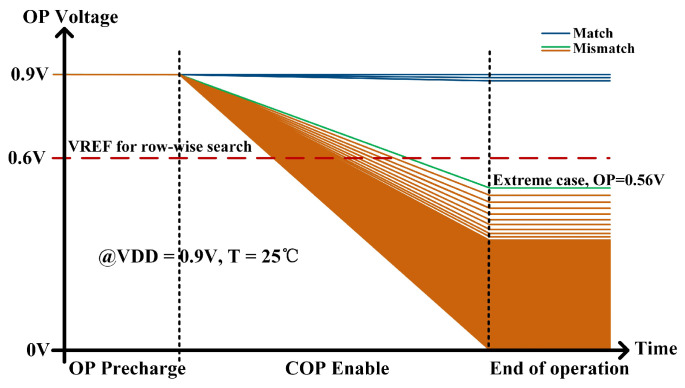
OP voltage of row-wise search operation through transient simulations.

The OP discharge for all cases is shown in Figure 10. In the most extreme scenario, where the number of search bits is 64 and the OP features 63 charging paths alongside only 1 discharging path, the discharge voltage of OP measures 0.56 V under the conditions of VDD = 0.9 V and T = 25 °C. For the remaining instances of matching failure, the voltage of OP falls below 0.56 V. Therefore, the search may be completed by simply identifying the OP level above 0.56 V using the auxiliary circuit shown in Figure 4.

#### 3.3.2. Column-Wise CAM Operation

In contrast to row-wise CAM, column-wise CAM requires two clock cycles. In the first cycle, when the control signal is activated, all COPs are pre-charged, and all ROPs are pre-discharged. Subsequently, the BL is pre-charged and employed as a match line, while OP is designated as a search line. After the configurations of signals, the ROPs corresponding to the selected rows undergo a charging process, initiating the search operation. The second cycle is similar to the first, with the exception that the BLB pre-charge functions as the match line, and the OP serves as the data line, loading the inverted results of the search data.

**Figure 11:**
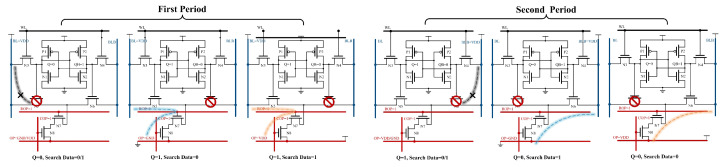
The RC-SRAM bitcell state of the column-wise search operation.

Figure 11 shows the different states of the bitcell in the column-wise search process. During the first cycle, when Q is 0, N5 is off and no path can be formed between BL and OP; when Q is 1 and the search data are 0, BL will form a discharging path with OP through N5, N7, and N8; and when Q is 1 and the search data are also 1, BL will form a charging path with OP through N5, N7 and N8. The second cycle is similar to the first.

**Figure 12:**
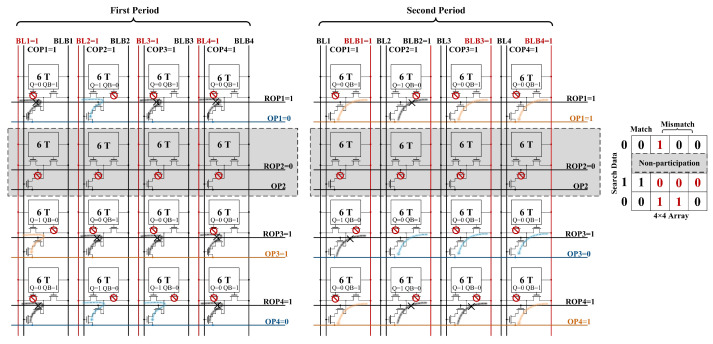
Example of column-wise search operation in 4 × 4 array.

As illustrated in Figure 12, the proposed column-wise search process is demonstrated using a simplified 4 × 4 array. In the context of the customized search operation, rows 1, 3, and 4 are designated for the search process, while row 2 is not involved in the search. Therefore, ROP2 is set to 0, indicating that it is not part of the search operation, and the remaining ROPs are pre-charged in preparation. The first cycle filters out the presence of columns with Q = 1 and Search Data = 0, such as the second and third columns. Subsequently, the second cycle filters out the presence of columns with Q = 0 and Search Data = 1, such as the fourth column. This two-cycle process comprehensively completes the column-wise search.

**Figure 13:**
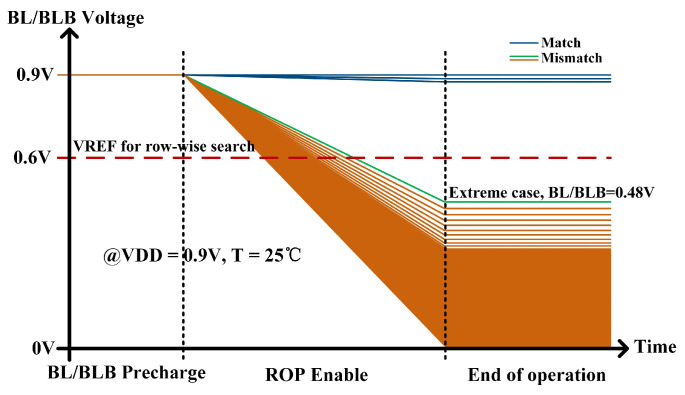
BL/BLB voltage of column-wise search operation through transient simulations.

At least one path to GND exists for the BL/BLB corresponding to the columns that do not match during the search. As depicted in Figure 13, in the context of the most extreme scenario where there are 16 search bits, BL/BLB forms 15 charging paths and just 1 discharging path. Under these conditions, with VDD of 0.9 V and T of 25 °C, the BL/BLB discharges to 0.48 V. For the remaining instances of matching failure, the voltage of OP falls below 0.48 V. Successful matching occurs when all paths of the BL/BLB are fully connected VDD. Similarly, the correctness of the column-wise search can be ensured by employing the auxiliary circuit depicted in Figure 4, which utilizes the INV with a threshold voltage of 2/3 VDD.

## 4. Simulation Results

In this section, we perform HSPICE simulations of the entire circuit on a 28 nm CMOS process with a supply voltage of 0.9 V and an ambient temperature of 25 °C to obtain and analyze the delay and power consumption for each operation.

**Figure 14:**
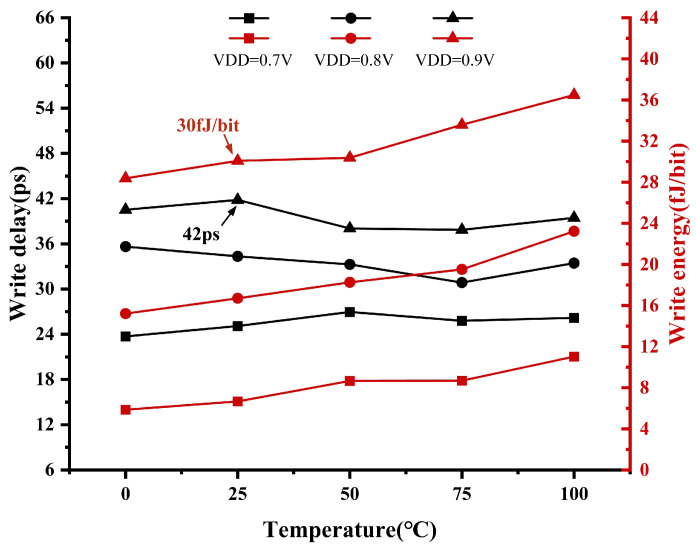
Simulation of write delay and energy across temperature and VDD.

In SRAM mode, the delay and energy of write operations versus the temperature and supply voltage traversal simulation results are plotted as shown in Figure 14. Write delay is defined as the time elapsed between the rise in the Word Line (WL) to half of the supply voltage (1/2 VDD) and the completion of the establishment of the storage node voltage. Notably, the simulation results demonstrate a low write delay, indicative of efficient performance. Temperature exerts a minor influence on the write delay but exerts a significant impact on power consumption during the write operations. As temperature increases, so does the energy consumption. Furthermore, the supply voltage has a discernible effect on both the write delay and energy, with a lower supply voltage resulting in reduced energy consumption and shorter write delay. At T = 25 °C and VDD = 0.9 V, the write delay is 42 ps, and the energy consumption is 30 fJ/bit.

**Figure 15:**
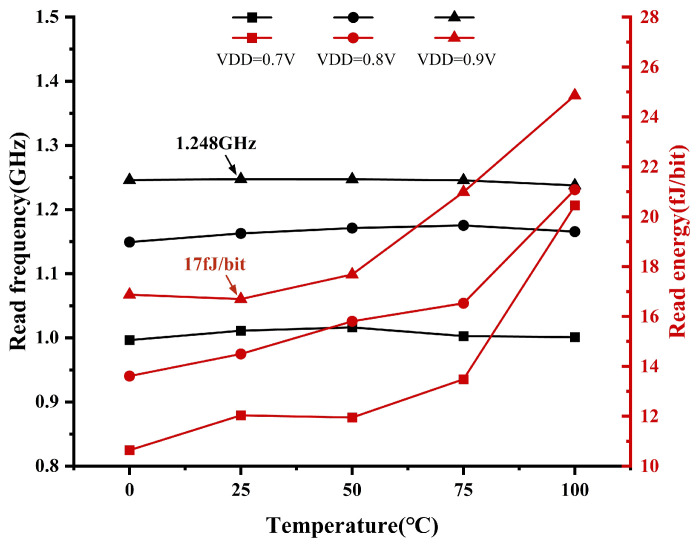
Simulation of read frequency and energy across temperature and VDD.

Figure 15 shows the simulation data analysis graph of the read operation frequency and energy consumption. The read operation time is defined as the time from when the read enable signal is valid until the circuits output the data. The simulation findings demonstrate that the temperature has a marginal impact on the read frequency and a huge influence on the read energy, which increases as the temperature rises. Furthermore, when the supply voltage is raised, there is a significant increase in both the read frequency and read power consumption. At T = 25 °C and VDD = 0.9 V, the read frequency is 1.248 GHz, and the energy consumption is 17 fJ/bit.

**Figure 16:**
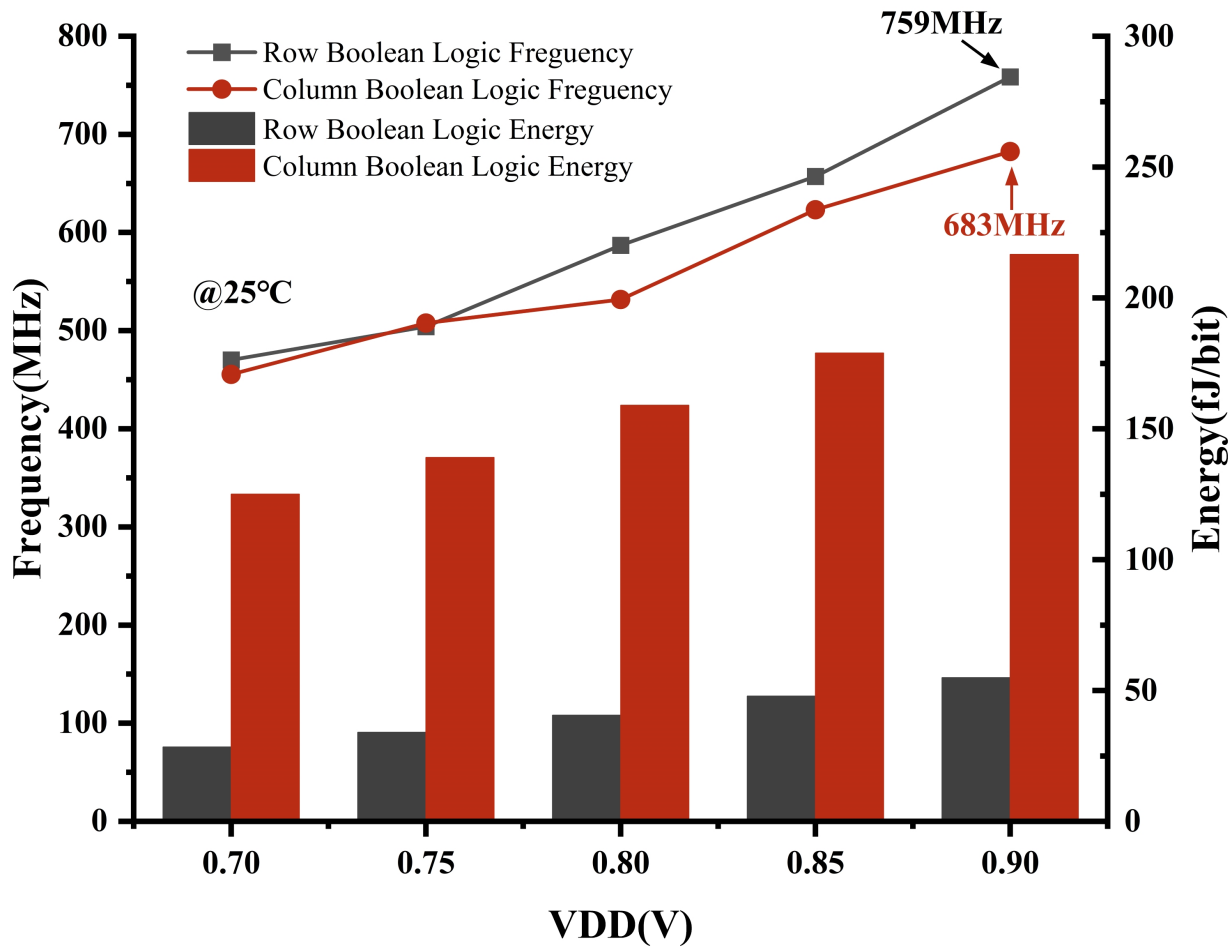
Simulation of Boolean logic frequency and energy across VDD at 25 °C.

As shown in Figure 16, for the Boolean logic mode, we simulate and analyze the frequency and energy consumption of bi-directional 2-bit operations for different VDD cases at 25 °C. The time for a Boolean logic operation is defined as the beginning of the enable signal to the output of the Boolean operation results, encompassing the entire process of signal configuration. As the voltage supply increases, it is expected that the frequency of Boolean logic operations will increase and so will the energy consumption. It is observed that column-wise Boolean operations consume more energy per bit because they participate in fewer bits of the operation, resulting in energy generated during the signal configuration process being averaged over fewer bits. By increasing the number of bits involved in Boolean logic operations, the energy consumption per bit can be effectively reduced. At T = 25 °C and VDD = 0.9 V, the row-wise Boolean logic has a frequency of 759 MHz and an energy consumption of 55 fJ/bit, and the column-wise Boolean logic has a frequency of 683 MHz and an energy consumption of 217 fJ/bit.

**Figure 17:**
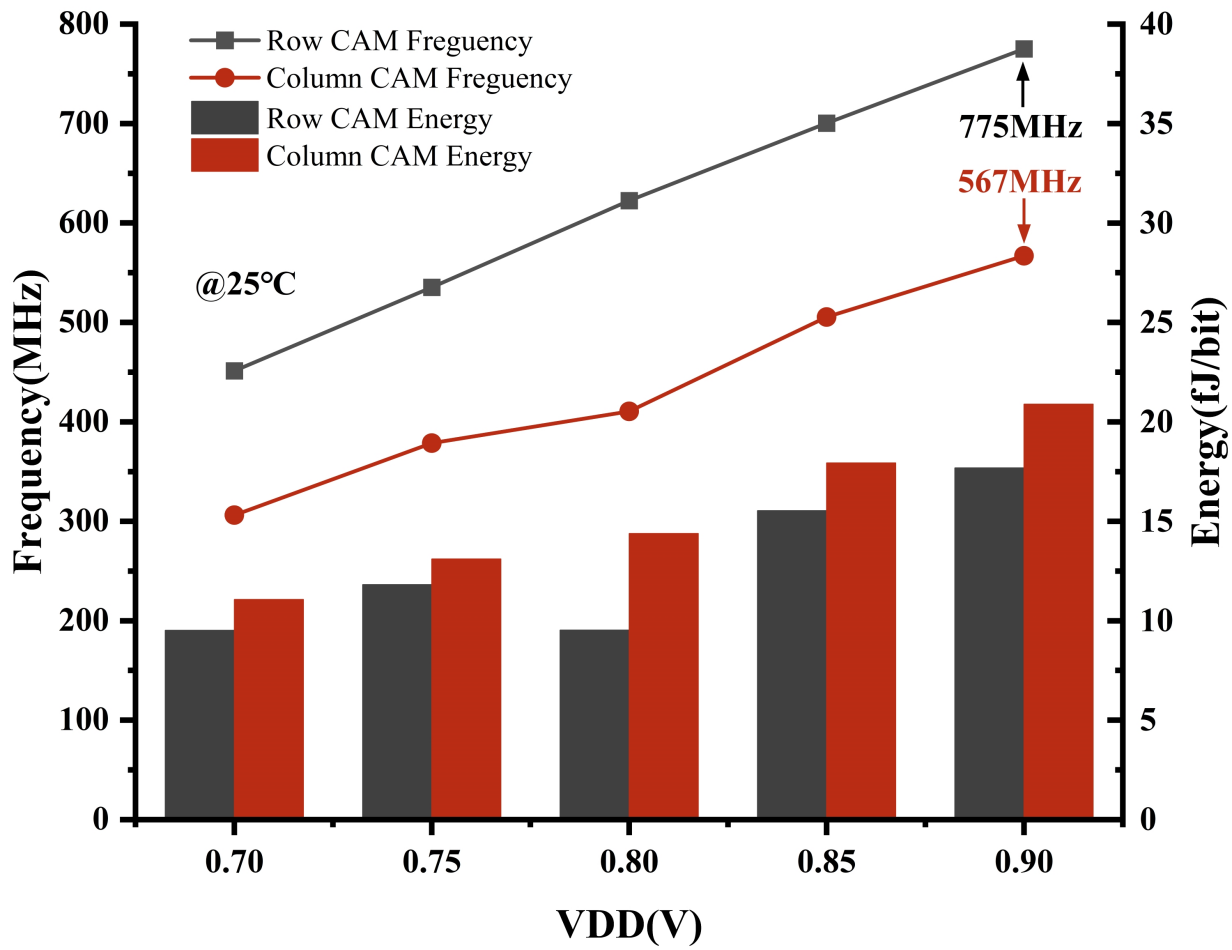
Simulation of CAM frequency and energy across VDD at 25 °C.

We simulate and analyze the frequency and energy consumption of a full array CAM search conducted row-wise and column-wise. Half-mismatch data are used for energy simulation, and one bit of mismatch data is used for frequency. The time for a CAM operation is defined as the beginning of the enable signal to the output of the search results. In Figure 17, it is evident that an increase in VDD results in a gradual increment in both the energy consumption and frequency. At T of 25 °C and VDD of 0.9 V, the row-wise CAM search operation has a frequency of 775 MHz and an energy consumption of 17.7 fJ/bit, and the column-wise CAM operation has a frequency of 567 MHz and an energy consumption of 20.9 fJ/bit. Similarly, by increasing the size of the array, the energy per bit can be effectively reduced.

Table 1 presents a comparison between the current study and previous research. It reveals that [19] solely supports the four-input unidirectional Boolean logic operation, while its CAM search merely allows for full-array unidirectional searching. The same holds true for [18], where the Boolean logic operation only supports two inputs, and the CAM method is limited to row-by-row search for array. Lin, Z. uses a special 10T SRAM to support row and column bi-directional Boolean logic operations and CAM search [15]. However, its CAM search is not operant controllable, and both row-wise OR/NOR and column-wise AND/NAND operations require an additional self-terminating structure to achieve bit customization, leading to an expansion in the circuit area. In terms of operating frequency, the Boolean logic and CAM search operating frequency of this work is comparable to that of [19], which is much higher than that of [15,18], providing excellent performance.

## 5. Conclusions

We propose a RC-SRAM bitcell for logic-in-memory and CAM search operations. This cell uses its special structure and different signal configuration methods that allow the implementation of both bi-directional and operand-controllable IMC. In addition, the use of threshold-controllable logic gates instead of the traditional SAs and ADCs sensing method effectively ensures the accuracy of the outputs and reduces the circuit area. The simulation results show that at a supply voltage of 0.9 V and an ambient temperature of 25 °C, the search speeds for the row and column bi-directional CAM operations achieve 775 MHz and 567 MHz, and the speeds for the row and column bi-directional full-Boolean logic operations reach 759 MHz and 683 MHz. These results demonstrate a significant increase in operating frequency, coupled with enhanced functionality that is not only more comprehensive but also highly adaptable to various situations and requirements.

## Figures and Tables

**Table 1:** Comparisons with other works.

			This Work	JSSC’16 [18]	JSSC’21 [15]	TVLSI’20 [19]
Technology			28 nm	28 nm FDSOI	28 nm	65 nm
Cell type			10 T	6 T	10 T	8 T
Array size			1 Kb	4 Kb	4 Kb	16 Kb
Supply voltage			0.9 V	1 V	0.9 V	1.2 V
Boolean logic	Row-wise	Freq.(MHz)	759	594	∼300	793
		Customizable ^*a*^	Yes	No	Yes	No
	Column-wise	Freq.(MHz)	683	NA	∼300	NA
		Customizable ^*a*^	Yes		Yes	
CAM search	Row-wise	Freq.(MHz)	775	NA	262	NA
		Customizable ^*b*^	Yes		No	
	Column-wise	Freq.(MHz)	567	∼370	256	813
		Customizable ^*b*^	Yes	No	No	No

^*a*^ signifies the capability to customize operands. ^*b*^ signifies the capability to customize involved rows or columns.

## Data Availability

Data are contained within this article.
